# Effects of myenteric denervation on extracellular matrix fibers and mast cell distribution in normal stomach and gastric lesions

**DOI:** 10.1186/1475-2867-10-18

**Published:** 2010-06-22

**Authors:** Cássia F Estofolete, Carla Botelho-Machado, Sebastião R Taboga, Sérgio Zucoloto, Ana Cláudia Polli-Lopes, Cristiane D Gil

**Affiliations:** 1 Department of Anatomy, School of Medicine - FAMERP, Avenida Brigadeiro Faria Lima 5416, CEP 15090-000, São José do Rio Preto, SP, Brazil; 2 Department of Biology, Institute of Biosciences, Humanities and Exact Sciences, IBILCE-UNESP, Rua Cristóvão Colombo 2265, CEP 15054-000, São José do Rio Preto, SP, Brazil; 3 Department of Pathology, School of Medicine, FMRP-USP, Avenida Bandeirantes 3900, CEP 14049-900, Ribeirão Preto, SP, Brazil

## Abstract

**Background:**

In this study the effect of myenteric denervation induced by benzalconium chloride (BAC) on distribution of fibrillar components of extracellular matrix (ECM) and inflammatory cells was investigated in gastric carcinogenesis induced by N-methyl-N'-nitro-N-nitrosoguanidine (MNNG). Rats were divided in four experimental groups: non-denervated (I) and denervated stomach (II) without MNNG treatment; non-denervated (III) and denervated stomachs (IV) treated with MNNG. For histopathological, histochemical and stereological analysis, sections of gastric fragments were stained with Hematoxylin-Eosin, Picrosirius-Hematoxylin, Gomori reticulin, Weigert's Resorcin-Fuchsin, Toluidine Blue and Alcian-Blue/Safranin (AB-SAF).

**Results:**

BAC denervation causes an increase in the frequency of reticular and elastic fibers in the denervated (group II) compared to the non-denervated stomachs (group I). The treatment of the animals with MNNG induced the development of adenocarcinomas in non-denervated and denervated stomachs (groups III and IV, respectively) with a notable increase in the relative volume of the stroma, the frequency of reticular fibers and the inflammatory infiltrate that was more intense in group IV. An increase in the frequency of elastic fibers was observed in adenocarcinomas of denervated (group IV) compared to the non-denervated stomachs (group III) that showed degradation of these fibers. The development of lesions (groups III and IV) was also associated with an increase in the mast cell population, especially AB and AB-SAF positives, the latter mainly in the denervated group IV.

**Conclusions:**

The results show a strong association in the morphological alteration of the ECM fibrillar components, the increased density of mast cells and the development of tumors induced by MNNG in the non-denervated rat stomach or denervated by BAC. This suggests that the study of extracellular and intracellular components of tumor microenvironment contributes to understanding of tumor biology by action of myenteric denervation.

## Background

Interaction between tumor cells and the surrounding stroma is one of the key aspects in the mechanism of tumor cell proliferation and invasion [1]. Tumor cells do remodel the extracellular matrix (ECM), a complex mixture of fibers (collagen, reticular and elastic) and ground substance that provides cell support [2], to facilitate communication and escape of the control by the microenvironment [3]. The collagen fibrillar system acts as a supporting framework of tissues, where reticular fibers connect collagen fibers with the basal laminae of epithelial, muscle and adipose cells; the microfibril-elastin system plays a role in uniformly distributing stress to maintain the resilience to local tissue requirements [4].

Structural and functional integrity of the collagen fibrillar and microfibril-elastin systems are important for the stomach to pack the food, to secrete enzymes and acids to break the raw nutrients and to transfer the mixture to the small intestine. The three tasks depend of the extrinsic (sympathetic and parasympathetic divisions of the autonomous nervous system) and the intrinsic innervation (enteric nervous system - ENS). The principal components of the ENS are two networks or plexuses of neurons and nervous fibers, the myenteric and submucosal plexus [5]. The ENS importance for the regulation of the gastrointestinal functions is observed after the topical application of the cationic surfactant, benzalkonium chloride (BAC), on the serous layer that results in partial and selective destruction of myenteric plexus neurons [6]. The correlation between carcinogenesis and the ENS has been demonstrated in experimental models using the myenteric denervation by BAC and the induction of tumors by N-methyl-N'-nitro-N-nitrosoguanidine (MNNG) [7] and 1,2-dimethylhydrazine (DMH) [8] with a reduction in the incidence and size of gastrointestinal tumors.

Immune cells are potent sources of paracrine signals to the ENS. Particularly enteric mast cells are strategically located and have powerful pharmacological mediators that act in the face of immunological stimuli that can affect the integrity of the gastrointestinal tract [9]. The antibody binding to mast cells makes them able to recognize specific antigens and signal their presence to ENS. ENS, in turn, interprets the chemical signals of mast cells as a threat and seeks to eliminate it, thus providing a protective response [9]. The presence of mast cells has been described in several neoplasias, with pro or anti-tumor roles played by their bioactive mediators released by the influence of the tumor microenvironment [10-12]. The action of pro-tumor mediators such as histamine, tryptase and chymase may promote migration and cell proliferation inducing the expression of adhesion molecules on endothelial cells and thus activating the process of tumor angiogenesis, metastasis and proliferation [13-15]. On the other hand, some cytokines such as interleukin (IL) -2 and -21, tumor necrosis factor (TNF) and heparin released by mast cells, can act as anticancer agents by inhibiting their growth [16,17].

Several studies have shown that during carcinogenesis there is an increase in the number of mast cells, observed in neurofibromas, lipomas, hemangiomas, tumors of the adrenal gland and skin [18], squamous cell carcinomas [19], laryngeal squamous cell [20] and gastric carcinomas [21]. In gastric adenocarcinomas, the small release of the contents stored in cytoplasmic granules of mast cells was associated with changes in the microvascular basal laminae, including irregular thickness, multiple layers and a weak association with endothelial cells and pericytes that contribute to the remodeling of blood vessels [22]. After surgical removal of gastric cancer, survival studies showed that patients with increased number of mast cells showed a worse prognosis compared to patients with low numbers of them.

The purpose of the present study was to determine the effects of chemical ablation of myenteric neurons on the distribution of the extracellular matrix fibers and mast cells in the gastric mucosa of non-denervated and denervated stomachs, and in a model of gastric carcinogenesis induced by MNNG administration in rats.

## Material and methods

### Experimental design

Four experimental groups were evaluated. Groups I (n = 5) and II (n = 5) non-denervated and denervated respectively, without the presence of gastric neoplasms; groups III (n = 10) and IV (n = 10) non-denervated and denervated, respectively, with the presence of gastric neoplasms. Fragments of the pyloric region (antrum) of stomach used in this study were obtained from investigations carried out by POLLI-LOPES et al. [7]. The experimental procedures were performed in accordance with the rules of Committee on Care and Uses of Laboratory Animals of the National Research Council of the N.I.H. (USA) and Ethics Committee for Animal Experimentation (CEEA) of FAMERP (Protocol n° 6193/2008), São José do Rio Preto, SP.

### Animals

Male *Wistar *rats weighting 100 a 150 g, obtained from the animal facility of the School of Medicine of Ribeirão Preto, Brazil, were housed (4 animals per cage), in temperatures between 23 to 25°C, and received food and water *ad libitum*.

### Gastric denervation

The animals were anesthetized i.m. with ketamine hydrochloride and thiazine chloride (0.15 ml/0.05 ml/100 g of weight) [23]. The stomach of each animal was exteriorized through a midline upper abdominal laparotomy and isolated from the peritoneal cavity through a small fenestration in a plastic sheet for topic application of 0.6% BAC (v/v) (Aldrich Chemical Co. diluted in saline (0.9% NaCl) [7,24]. The isolated stomachs were wrapped with gauze soaked in BAC or saline and kept moist for 30 min [6,7,24]. The serosal surface of the stomachs was thoroughly washed with saline, the organs were returned to their anatomical place and the abdominal wall was sutured. The animals were maintained in plastic cages (4 animals/cage) under controlled temperature, and received food and water *ad libitum*.

#### Induction of neoplasias in gastric antrum mucosa

Sixteen weeks after surgery, the animals of groups III (non-denervated) and IV (denervated) ingested a solution of N-methyl-N'-nitro-N-nitrosoguanidine (MNNG) (Aldrich Chemical Co., Inc., Milwaukee, WI) dissolved in distilled and deionized water at a concentration of 100 mg/L for 28 weeks. The animals were sacrificed 2 months after the last intake of MNNG and the stomachs were collected for morphological analysis.

### Morphological and quantitative analysis

#### Extracellular matrix fibers

Fragments of the pyloric region of the stomach (antrum) were fixed in 4% buffered formalin for 12 hours, washed in tap water, dehydrated in an ethanol series and embedded in paraffin. For the extracellular matrix study, sections 4 μm-thick were stained with Hematoxylin-Eosin [25], Picrosirius-Hematoxylin [26] and Gomori reticulin [27] for histopathological analysis, and for collagen and reticular fibers evaluation respectively. The fibers of elastic system were studied using Weigert's Resorcin-Fuchsin that selectively stains both elastic and elaunin fibers (the oxytalan fibers remain unstained because they are not previously oxidized in this study) [28]. The specimens were analyzed with an Olympus BX60 light microscope (Olympus, Hamburg, Germany) and the microscopic fields were digitalized using the Image-Pro Plus version 4.5 for Windows software (Media Cybernetics, Silver Springs, MD). Five random areas of the gastric mucosa were analyzed using an ×20 objective, and the calibration was done using an Olympus graded microscopic slide. The quantitative analysis was determined according to the procedure of Weibel [29], a grid test system with 168 points and 84 test lines, to compare the relative volume (percentage) of the gastric mucosa compartments (epithelium, stroma and others compartments, except the epithelium and stroma) for Hematoxylin-Eosin staining and the relative frequency of reticular and elastic fiber distribution (percentage) for Gomori reticulin and Weigert's resorcin- fuchsin staining, respectively. Values are reported as mean ± SEM of relative volume (percentage) of the gastric mucosal compartments and mean ± SEM of relative frequency of reticular and elastic fiber distribution (percentage).

#### Mast cells

Analysis of mast cells in the gastric antrum was performed in sections stained with Toluidine Blue 0.5% for quantification of intact and degranulated cells and, with Alcian-Blue-Safranin (AB-SAF) for quantification of mucosal and connective tissue mast cells [30,31]. For this purpose, sixty randomly areas of the gastric mucosa were analyzed, per animal, with an Olympus light microscope (Olympus, Hamburg, Germany) using a × 12,5/16 millimeter ocular and a × 40 power objective (ZEISS, Germany). Values are reported as mean ± SEM of the number of cells per mm ^2^.

### Statistical Analysis

Data were analyzed using Statistica 6.0 software (StarSoft, Inc., Tulsa, OK). The data are presented as mean ± SEM and were submitted to the Student's *t *test and one-way analysis of variances (ANOVA), and then to the Tukey-Kramer multiple comparisons test or Bonferroni. Differences with values of *P *< 0.05 were considered statistically significant.

## Results

### Histopathological and histochemical analysis of rat stomachs

**Figure 1:**
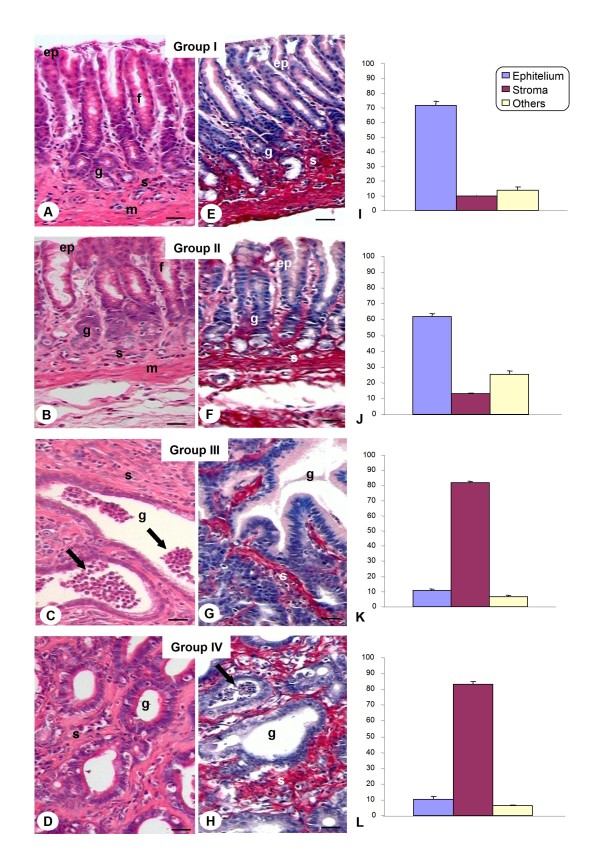
**Histological analysis of rat stomachs**. *A, B, E and F) *Pyloric gastric glands (g) surrounded by stroma with collagen fibers (s) observed in the mucosa layer of the groups I and II. Epithelium (ep). Gastric pits (f). Muscularis mucosa (m). *C, D, G and H) *Pyloric gastric glands (g) constituted by tumoral cells occupied the largest area of the gastric mucosa of the groups III and IV. An accumulation of PMN (arrows) was observed within the glands. Hematoxylin-Eosin (A-D); Picrosirius-Hematoxylin (E-H). Bars: 20 μm. *I-L*) Tissue compartments (epithelium, stroma, others) volumes of the gastric pyloric mucosa. Values are expressed as mean ± SEM of percentage of relative volume. Groups without lesions (I and II) presented a high relative volume of epithelium (p < 0.05) compared to groups with lesions (III and IV). The presence of adenocarcinoma in the groups III and IV resulted in a significant increase (p < 0.05) of stroma compared to groups I and II.

Initially, pyloric fragments of non-denervated and denervated stomachs by benzalkonium chloride (BAC) (groups I and II, respectively) were studied. Analysis of fragments of group II showed that myenteric denervation didn't change the quantity and distribution of the meshwork collagen fibers that surround the pyloric glands compared to group I (Fig. 1A and 1B). This result was confirmed by Picrosirius-Hematoxylin stain (Fig. 1E and 1F) and quantitative analysis of the relative volume of the epithelium and stroma of the gastric mucosa from both groups (Fig. 1I and 1J).

**Figure 2:**
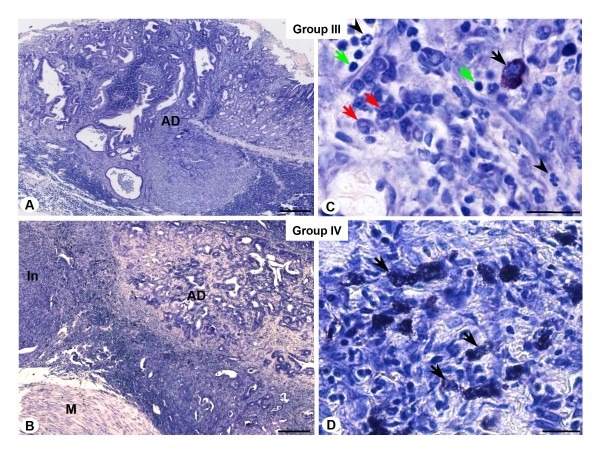
**Histological analysis of adenocarcinoma induced by MNNG treatment**. *A and B) *Morphological view of adenocarcinoma (AD) in non-denervated (group III) and denervated stomach (group IV). Muscular layer (M). Intense inflammatory infiltrate (In). *C and D) *Detail of inflammatory infiltrate of tumor stroma constituted by mast cells (black arrows), neutrophils (arrowheads), lymphocytes (green arrows) and plasma cells (red arrows). Toluidine Blue. Bars: *100 μm (Fig. A and B), 10 μm (Fig. C and D)*.

Non-denervated and denervated stomachs treated with MNNG (groups III and IV, respectively) presented changes in tissue architecture and arrangement of the fibers of the extracellular matrix and an increase of the diameter of pyloric glands as well as in relative volume of the stroma (Fig. 1C, D, G and 1H). Treatment with MNNG induced the development of benign and malign tumors, especially adenomatous polyps and adenocarcinomas (Fig. 1C and 1G) in non-denervated stomachs (group III). Animals with denervated stomachs and treated with MNNG (group IV) developed precancerous lesions and malign tumors characterized by dysplasia, atrophic gastritis and adenocarcinoma (Fig. 1D and 1H). In both groups, the stroma of the adenocarcinomas was enriched by the presence of inflammatory cells, as mast cells, neutrophils, plasma cells and lymphocytes, showing immunological response to neoplasias (Fig. 2). However, the neoplastic lesions of group IV were of smaller and less aggressive profiles when compared to group III, and the inflammatory infiltrate localized in the tumor stroma was more intense, especially in animals that developed adenocarcinomas (Fig. 2D).

**Figure 3:**
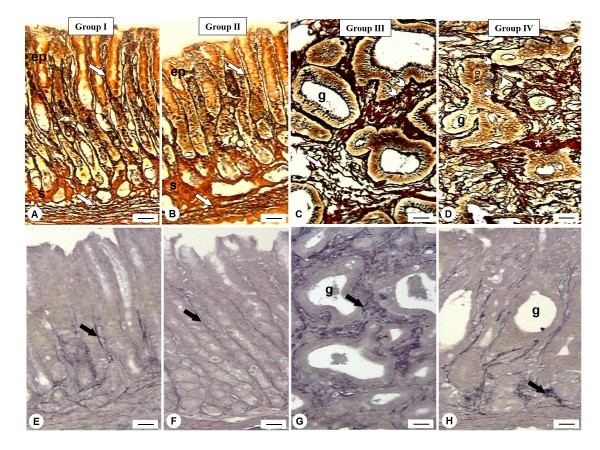
**Histochemical analysis of reticular (A-D) and elastic fibers (E-H) in the rat stomachs**. *A and B) *Groups I and II presented reticular fibers rectilinear and aligned (arrows) surrounding the epithelium (ep) and the stroma (s). *C and D) *Reticular fibers (arrow) winding and apparently densest at the base (arrowheads) of glands (g). Collagen fibers accumulation between the glands (*) were observed. *E and F) *The glands are accompanied by a delicate web of elastic fibers (arrow) and more intensely marked in E. *G and H) *An increase in the thickness of these fibers, disruption and fragmentation of fibrillar variables grouped masses of elastin (arrows) can be observed around the glands (g) compared to their respective control groups (I and II). Gomori's silver (A-D); Weigert's Resorcin-Fuchsin (E-H). Bars: *10 μm*.

Histochemical analysis of the pyloric sections was performed with Gomori reticulin and Weigert's Resorcin-Fuchsin staining to study the distribution (frequency) of the reticular and elastic fibers in the stroma, respectively. Sections submitted to both methods revealed similar distribution of the meshwork fibers that surround the pyloric glands of the stomachs from groups I and II (Fig. 3A, B, E and 3F). Non-denervated or denervated stomachs treated with MNNG (groups III and IV, respectively), apparently presented increased amount of reticular fibers (Fig. 3C and 3D) and thick and short elastic fibers highly disorganized (Fig. 3G and 3H) in the adenocarcinomas compared to their respective control groups (groups I and II, respectively).

Quantitative analysis of the reticular fibers confirmed the increase of this extracellular component in the adenocarcinomas observed in the groups III and IV and also showed an unexpected significant increase in the mucosa of denervated stomach (group II) compared to group I (Table 1). Despite the apparent increase of elastic fibers in the stroma of non-denervated and denervated animals treated with MNNG (groups III and IV, respectively), no statistical difference was observed in their distribution compared to control groups (I and II) (Table 1). On the other hand, the myenteric denervation induced significant increase of the elastic fibers of gastric stroma from groups II and IV compared to non-denervated stomachs from groups I and III (Table 1). No significant difference was noted in the collagen and elastic fiber distribution between non-malign and malign lesions of stromas from groups III and IV.

**Table 1:** Stereological evaluation of the effects of myenteric denervation in the gastric antrum.

	**Relative frequency distribution of the fibers(%)**^**1**^	
	
Groups	Collagen System	Elastic System
I	16.87 ± 0.5	13.27 ± 0.5
II	24.06 ± 1.5*	19.4 ± 0.6*
III - NT	21.8 ± 0.9	11.2 ± 0.6
III - MT	25.71 ± 0.5*	11.1 ± 0.4
IV - NT	21.2 ± 1.6	20.2 ± 1.7^&^
IV - MT	26.87 ± 1.5*	19 ± 0.7^&^

Histological preparation was performed as described in *Materials and Methods*. Values are expressed as mean ± SEM of percentage of relative frequency of fibers. Non-malign tumor (NT); malign tumor (MT). ^1 ^Statistical analysis based on ANOVA followed by the correction of Tukey-Kramer. *P < 0.05 vs. group I; ^&^P < 0.05 vs. group III.

### Quantification of mast cells in the fragments of the pyloric region of the stomach

**Figure 4:**
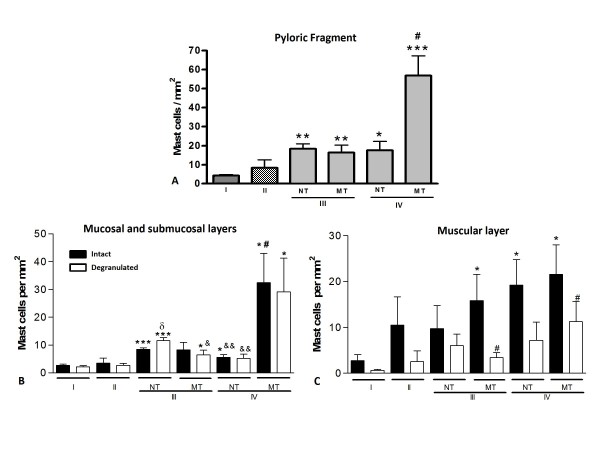
**Quantitative analysis of mast cells in the stomach**. Data represent the mean ± S.E.M of sixty fields analyzed per animal as described in Material and Methods. Non-denervated **(I) **and denervated stomach **(II) **without lesions. Non-denervated **(III) **and denervated stomach **(IV) **with lesions. Non-malignant tumor **(NT)**. Malignant tumor **(MT)**. *A) *Total number of mast cells in the gastric fragment. *P < 0.05 vs. group I. # P < 0.01 vs. another experimental groups. *B and C) *Intact and degranulated mast cells in the mucosal, submucosal and muscular layers. * P < 0.05 vs. group I; #P < 0.05 vs. another experimental groups; δ P < 0.05 vs. group III - NT (intact mast cells); &P < 0.05 vs. group III - NT (degranulated mast cells). *C) *Intact and degranulated mast cells in the muscular layer. *P < 0.05 vs. group I; # P < 0.05 vs. group II.

Mast cells were analyzed and quantified in the mucosal, submucosal and muscular layers in all experimental groups. The analysis of the total number of cells per gastric fragment showed no significant difference between the non-denervated (I) and denervated (II) groups (Fig. 4A). However, in non-denervated or denervated groups treated with MNNG (III and IV, respectively), the development of benign or malignant lesions was associated with a significant increase of the number of mast cells in the gastric antrum compared with the non-denervated group (I). In the mucosal and submucosal layers, the significant increase of mast cells in the non-denervated or denervated groups treated with MNNG, (III and IV) was associated with an increase of intact and degranulated cells (Fig. 4B). In the muscle layer, this increase was mainly associated with the large number of intact mast cells (Fig. 4C).

### Phenotypic analysis of the mast cells

**Figure 5:**
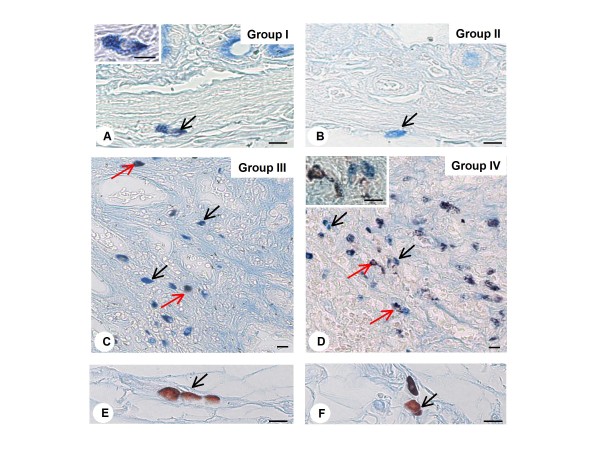
**Phenotypical analysis of mast cells of gastric fragment**. *A and B) *Submucosal layer of non-denervated (group I) and denervated (group II) stomach showing mast cells with cytoplasmic granules positive to alcian-blue (arrows), characterizing mucosal mast cell phenotype - in detail of panel A. *C and D) *Two mast cell population are noted in the gastric lesions of non-denervated (group III) and denervated (group IV) stomachs: alcian-blue positive mast cells (black arrows) and alcian-blue/safranin positive mast cells (red arrows). *E and F) *Mast cells with safranin positive granules (arrows) are observed in the mesentery, characterizing connective tissue mast cells. Alcian-blue/Safranin method. *Bars: 10 μm (Fig. A-F); 5 μm (detail Fig. A)*.

For the phenotypic characterization of mast cells it was used the method of Alcian Blue-Safranin (AB-SAF). In animals of non-denervated (I) and denervated (II) groups, there was a predominance of mast cells AB positive (AB^+ ^) in the mucosal and submucosal layers of the gastric antrum, characterizing these cells as mucosal mast cells (MMC) (Fig. 5A and 5B). In the experimental groups treated with MNNG (III and IV), a high proportion of AB^+ ^and AB-SAF^+ ^mast cells subtypes were observed, the latter mainly in the denervated group (IV) (Fig. 5C and 5D). Fragments of the gastric antrum containing mesentery were used to confirm the presence of SAF^+ ^cells, characterizing the connective tissue mast cell (CTMC) by this method of staining (Fig. 5E and 5F).

The total quantification of three subtypes of mast cells (AB^+ ^, AB-SAF^+ ^and SAF^+ ^) confirmed the morphological observations, showing a predominance of AB^+ ^cells in the pyloric fragment in all experimental groups (Table 2). In non-denervated and denervated groups treated with MNNG (III and IV, respectively), this cell subtype increased significantly compared to the non-denervated group (I). In denervated stomachs treated with MNNG (group IV), sub-cellular AB-SAF^+ ^also was significantly increased in malignant lesions compared to lesions in the non-denervated group treated with MNNG (III).

**Table 2:** Density of mast cells with different phenotypes in the gastric antrum.

	Pyloric Fragment		
	
Groups	AB+	SAF+	AB+ SAF+
I	8 ± 2	1 ± 1	4 ± 1
II	14 ± 4	3 ± 1	13 ± 10
III - NT	49 ± 16^*^	5 ± 2	11 ± 4
III - MT	23 ± 4^***^	4 ± 2	13 ± 5
IV - NT	28 ± 5^***^	2 ± 1	23 ± 8
IV - MT	36 ± 8^**#^	13 ± 10	47 ± 12^&^

Histological preparation was performed as described in *Materials and Methods*. Values (number of cells per mm^2^) are expressed as mean ± SEM of 60 fields analysed per animal with a high power objective (40×). *Alcian-blue *(AB+), safranin (SAF+) and *alcin-blue*/safranin positive cells (AB+SAF+). ^*^ P < 0.05 vs. group I; ^#^ P < 0.01 vs. III - NT and - MT; ^&^ P < 0.05 vs. II.

The analysis of the different subtypes of mast cells in the mucosal and submucosal layers of pyloric fragments showed a significant increase of mast cells AB^+ ^only in animals with a benign tumor (Table 3). In denervated stomachs treated with MNNG (group IV), the population of mast cells AB-SAF^+ ^increased in comparison with other experimental groups, being significant for malignant neoplasm. The muscle layer showed a different profile of the fragment as a whole, and denervated stomachs without and with neoplasia (groups II and IV) showed a predominance of mast cells AB-SAF^+ ^. In neither group studied, the number of cells SAF^+^, which characterize the CTMC, overtook the other cell subtypes.

**Table 3:** Distribution of mast cells with different phenotypes in the layers of gastric antrum.

	Histological Layers					
	
	Mucosa and Submucosa			Muscular		
	
Groups	AB+	SAF+	AB+ SAF+	AB+	SAF+	AB+ SAF+
I (Control)	7 ± 2	0 ± 0	3 ± 0	1 ± 0	1 ± 1	2 ± 1
II	12 ± 4	0 ± 0	2 ± 1	2 ± 1	2 ± 1	11 ± 9
III - NT	40 ± 14^*^	1 ± 1	4 ± 1	8 ± 3^*^	3 ± 2	7 ± 3
III - MT	12 ± 2	0 ± 0	3 ± 1	11 ± 3^*^	4 ± 1	10 ± 4
IV - NT	19 ± 3^**^	1 ± 0,6	12 ±8	9 ± 3^*^	1 ± 0	18 ± 7
IV - MT	27 ± 10	9 ± 8	25 ± 8^#&^	9 ± 2^**π^	3 ± 2	22 ± 9

Histological preparation was performed as described in *Materials and Methods*. Values (number of cells per mm^2^) are expressed as mean ± SEM of 60 fields analysed per animal with a high power objective (40×). *Alcian-blue *(AB+), safranin (SAF+) and *alcin-blue*/safranin positive cells (AB+SAF+). ^*^ P < 0.05 vs. group I; ^#^ < 0.05 vs. I and III-MT; ^&^ P < 0.01 vs. II; ^π^ P < 0.05 vs. II.

## Discussion

In this study was investigated the histopathological changes and distribution of the extracellular matrix (ECM) fibrillar components, and mast cells in the pyloric region of rat stomachs non-denervated or denervated by benzalkonium chloride (BAC) and, non-treated or treated with the carcinogen N -methyl-N '-nitro-N-nitrosoguanidine (MNNG).

Initially, the histopathological and stereological analysis showed no changes in the relative volume of the epithelial and stromal compartments in the pyloric fragments from non-denervated (group I) and denervated stomachs (group II). However, the study of fibrillar components of ECM in these specimens revealed that BAC denervation causes an increase in the frequency of reticular and elastic system fibers in the denervated gastric mucosa (group II) compared to non-denervated (group I), as demonstrated by histochemical and stereological analysis using Gomori reticulin and Weigert's Resorcin-Fuchsin staining, respectively. Probably, the absence of contractile stimulation caused by myenteric denervation in these fragments (group II) contribute to an increase in the synthesis of reticular and elastic fibers by smooth muscle cells and/or a new association of fibers in the stroma as a mechanism to adequate the peristaltic wave coordination, and to prevent stasis in denervated organs [32-34].

Treatment of non-denervated (group III) and denervated (group IV) animals with MNNG induced the development of malignant tumors (adenocarcinomas), benign tumors (adenomatous polyps) and precancerous lesions (dysplasia and atrophic gastritis). The development of adenocarcinomas in both non-denervated and denervated stomachs was able to promote a significant increase in the relative volume of the stromal compartment. During the tumor development in the prostate or gastric mucosa, the interaction stroma/epithelium is disrupted and imposed a new condition associated to the morphologic changes of extracellular matrix components and tumor growth [35]. The epithelial cell proliferation in both malignant and benign lesions causes changes located in the gland, followed by a focal remodeling of the fibrillar components in the extracellular matrix [27].

In this aspect, the histochemical analysis showed a significant increase on the reticular fibers of the adenocarcinoma (group III) compared to the non-denervated stomachs without lesions (group I). The increase of stromal collagen fibers represents a mechanism by which tumor cells could escape the "attack" of T lymphocytes (CD8^+ ^) and to induce apoptosis, that would act as a barrier to the infiltration of CD8^+ ^[36]. Despite a not significant alteration on the elastic system, a disruption of these fibers was observed in adenocarcinomas of the non-denervated stomach (group III), with the presence of bodies of elastic elements fractionated, suggesting a degradation or remodeling of the stroma in response to injury under the influence of neoplastic cells. These changes in elastic system fibers were simultaneous with the remodeling of collagen fibers, also observed in prostatic lesions [37] and in the prostatic stroma during this gland regression following castration [38,39]. According to these results, the aggressive tumor could be evaluated by the fibrous components of the matrix. A more developed mesh of collagen fibers found in neoplastic lesions benign or malignant of non-denervated stomachs associated with degradation of elastic fibers would be responsible for an appropriate support that ensures the success of tumor growth.

On the other hand, in the denervated stomach with adenocarcinoma (group IV), the increase of stroma was not accompanied by increased of reticular and elastic fiber compared to denervated stomachs without lesion (group II), suggesting that the myenteric denervation prior to the process of carcinogenesis could protect the gastric mucosa from the development of the lesion. The precancerous and neoplastic lesions of denervated stomachs by BAC also showed a web of reticular and elastic system fibers without degradation probably synthesized prior to the installation of the lesion; this situation may have contributed to a smaller development of gastric lesions.

In addition to morphological changes of ECM fibrillar components, development of adenocarcinomas in the denervated stomach (group IV) was associated with inflammatory infiltrate most intense in the stroma in relation to the stomach without denervation (III). This exacerbation of the inflammatory response could be attributed to anti-inflammatory action of the nervous system that is able to gather information about inflammatory events in various locations, mobilizing defenses and thus contributing to a more efficient immune memory [40].

The parasympathetic nervous system, which includes the vagus nerve, is recognized as a powerful agent in neuroimmune inflammation of the digestive system [41]. Its anti-inflammatory action seems to be related to its main neurotransmitter, acetylcholine, capable of inhibiting the activation of immune cells by binding to its receptors on monocytes and macrophages [42,43], dendritic cells [44] and mast cells [45,46].

Knowing that inflammation is also involved in the process of tumorigenesis by regulating the growth, migration and differentiation of cells in the tumor microenvironment through the action of inflammatory cells [47], the next step of this study was to characterize the distribution of these cells, particularly mast cells in this experimental model. The pathological examinations revealed no changes in the number of mast cells between the non-denervated (I) and denervated (II) gastric antrum. However, the gastric lesions induced by MNNG in the experimental groups with and without myenteric denervation (III and IV) showed a significant increase in the number of mast cells in the non-denervated group (I). Moreover, the morphological study of mast cells demonstrated the presence of intact and degranulated cells in similar proportions in the mucosal and submucosal, region of tumors, in both experimental groups. The increase in the density of mast cells has been linked to several types of cancer such as melanoma, ovarian cancer, head and neck carcinomas, breast, lung [10-12] and gastric cancers [14,21,22,48]. The action of mast cell can be pro or anti-tumor depending on the mediators released into the microenvironment [10-12]. Among these mediators, stand out pro-angiogenic factors, such as heparanase and vascular endothelial growth factor (VEGF) and tryptase and chymases that provide tumor growth. Moreover, some cytokines (IL-2, IL-21 and TNF) and heparin can act as anti-tumor mediators, released by mast cells, limiting tumor progression [10].

The study of phenotypic characterization of mast cells was based on the method of Alcian Blue-Safranin [21,30] to classify them as mucosal (MMC) and connective tissue mast cells (CTMC). In animals of non-denervated (I) and denervated (II) groups there was a predominance of MMC in the mucosal and submucosal layers of gastric antrum confirming published data [49,50]. In non-denervated or denervated stomachs treated with MNNG (groups III and IV), this cell subtype increased significantly compared to the group I. Beyond MMC, the denervated gastric antrum of animals treated with MNNG (IV) showed a significant increase in other cell subtype alcian blue-safranin positive, suggesting a phenotypic change of MMC to CTMC associated with denervation. This high density of CTMC was observed mainly in the muscular layer of the antrum, the region most affected by myenteric denervation [7]. Osinski and colleagues [51] also described a phenotypic change of mast cells in the jejunum muscular layer of rats following myenteric denervation, revealed by the fluorescence of berberine sulfate in the cytoplasmic granules of mast cells. According to the phenotypes, mast cells may mediate immunosuppression, contributing to a more efficient immune tolerance [11]. The cytoplasmic granules of CTMC contain heparin which has an anti-tumor role demonstrated by studies using the implant in mice 4T1 cells (cell line of mammary adenocarcinoma) treated or not with imatinib, a drug that induces cell death [52]. The animals that received the implant of cells treated with imatinib developed larger tumors compared to tumors in animals implanted with untreated cells. In the same study, the researchers showed that mice deficient for the heparin synthesis showed an increase in tumor growth, suggesting a lowering effect of this mediator in tumor development. Thus, the protective effect of myenteric denervation on the development of tumors in rats could also be attributed to phenotypic changes of mast cells from MMC to CTMC, contributing to the release of anti-tumor mediators.

## Conclusions

The morphological aspects discussed herein, the extracellular and intracellular components of tumor microenvironment, are of fundamental importance for understanding the tumor biology by action of myenteric denervation and the establishment of tissue phenotypes in pathological conditions. Thus, research on pathological examination in complex biological models for the study of mast cells and fibrous proteins of the ECM as a target for new therapies in the tumorigenesis, are essential and should have direct clinical implications.

## Competing interests

The authors declare that they have no competing interests.

## Authors' contributions

ACPL carried out the experimental design. SZ was responsible for histopathology and classification of gastric lesions. CFE and CBM carried out the morphological and statistical analysis of extracellular matrix fibers and mast cells. SRT performed the stereological analysis. CDG and ACPL conceived and coordinated the study. CFE, ACPL and CDG wrote the manuscript. All authors read and approved the final manuscript.

## References

[B1] Pietras K Ostman A Hallmarks of cancer: Interactions with the tumor stroma Exp Cell Res 2010 316 8 1324 31 10.1016/j.yexcr.2010.02.045 20211171

[B2] Tanzer ML Current concepts of extracellular matrix J Orthop Sci 2006 11 3 326 331 10.1007/s00776-006-1012-2 16721539 PMC2778692

[B3] Pupa SM Ménard S Forti S Tagliabue E New insights into the role of extracellular matrix during tumor onset and progression J Cell Physiol 2002 192 3 259 267 10.1002/jcp.10142 12124771

[B4] Ushiki T Collagen fibers, reticular fibers and elastic fibers. A comprehensive understanding from a morphological viewpoint Arch Histol Cytol 2002 65 2 109 126 10.1679/aohc.65.109 12164335

[B5] Goyal RK Hirano I The enteric nervous system N Engl J Med 1996 334 17 1106 1115 10.1056/NEJM199604253341707 8598871

[B6] Sakata K Kunieda T Furuta T Sato A Selective destruction of intestinal nervous elements by local application of benzalkonium solution in the rat Experientia 1979 35 1611 1613 10.1007/BF01953222 520469

[B7] Polli-Lopes AC Zucoloto S Cunha FQ Figueiredo LAS Garcia SB Myenteric denervation reduces the incidence of gastric tumors in rats Cancer Lett 2003 190 45 50 10.1016/S0304-3835(02)00584-0 12536076

[B8] Garcia SB Oliveira JSM Pinto LZ Zucoloto S The relationship between megacolon and carcinoma of the colon: an experimental approach Carcinogenesis 1996 17 8 1777 1779 10.1093/carcin/17.8.1777 8761443

[B9] Wood JD Enteric neuroimmunophysiology and pathophysiology Gastroenterology 2004 127 2 635 657 10.1053/j.gastro.2004.02.017 15300595

[B10] Galinsky DST Nechushtan H Mast cells and cancer - No longer just basic science Crit Rev Oncol Hematol 2008 68 2 115 130 10.1016/j.critrevonc.2008.06.001 18632284

[B11] Wasiuk A Vries VC Hartmann K Roers A Noelle RJ Mast cells as regulators of adaptive immunity to tumors Clin Exp Immunol 2008 155 140 146 10.1111/j.1365-2249.2008.03840.x 19077084 PMC2675243

[B12] Maltby S Khazaie K Mcnagny KM Mast cells in tumor growth: Angiogenesis, tissue remodelling and immune-modulation Biochim Biophys Acta 2009 1796 1 19 26 19233249 10.1016/j.bbcan.2009.02.001 PMC2731828

[B13] Yano H Kinuta M Tateishi H Nakano Y Matsui S Monden T Okamura J Sakai M Okamoto S Mast cell infiltration around gastric cancer cells correlates with tumor angiogenesis and metastasis Gastric Cancer 1999 2 1 26 32 10.1007/s101200050017 11957067

[B14] Kondo K Muramatsu M Okamoto Y Jin D Takai S Tanigawa N Miyazaki M Expression of chymase-positive cells in gastric cancer and its correlation with the angiogenesis J Surg Oncol 2006 93 36 42 10.1002/jso.20394 16353179

[B15] Ozdemir O Can chymase-positive mast cells play a role in the progression of gastric cancer via angiogenesis? J Surg Oncol 2006 94 260 262 10.1002/jso.20570 16900528

[B16] Wang X Ruan Y Wu Z Studies of mast cells-mediated cytotoxicity to hepatoma cells in vitro Zhonghua Zongliu Zazhi 1996 18 276 278 9387320

[B17] Theoharides TC Conti P Mast cells: the Jekyl and Hyde of tumor growth Trends in Immunol 2004 25 235 241 10.1016/j.it.2004.02.013 15099563

[B18] Pastrnak A Jansa P Kolar Z Mastocytes in the process of cancerogenesis. I. Study of experimental model systems Cesk Patol 1986 22 4 210 213 3791452

[B19] Flynn EA Schwartz JL Shklar G Sequential mast cell infiltration and degranulation during experimental carcinogenesis J Cancer Res Clin Oncol 1991 117 2 115 122 10.1007/BF01613134 1901064 PMC12200042

[B20] Silistino-Souza R Rodrigues-Lisoni FC Cury PM Maniglia JV Raposo LS Tajara EH Christian HC Oliani SM Annexin 1: differential expression in tumor and mast cells in human larynx cancer Int J Cancer 2007 120 2582 2589 10.1002/ijc.22639 17340616

[B21] Liu YL Zhao MQ Hou G Miao F Liu W Effects of mast cell infiltration on the development and metastasis of gastric carcinoma Di Yi Jun Yi Da Xue Xue Bao 2005 7 809 811 10.3736/jcim20090902 16027074

[B22] Caruso RA Fedele F Zuccalá V Fracassi MG Venuti A Mast cell and eosinophil interaction in gastric carcinomas: ultrastructural observations Anticancer Res 2007 27 1A 391 394 17352258

[B23] Van Pelt LF Ketamine and xylazine for surgical anesthesia in rats J Am Vet Med Assoc 1977 171 842 844 924855

[B24] Sobreira LF Zucoloto S Garcia SB Troncon LE Effects of myenteric denervation on gastric epithelial cells and gastric emptying Dig Dis Sci 2002 47 11 2493 2499 10.1023/A:1020508009213 12452385

[B25] Behmer AO Tolosa EMC Freitas-Neto AGF Manual de Práticas para Histologia Normal e Patológica 1976 São Paulo: Edart-Edusp

[B26] Junqueira LCU Bignolas G Brentani R Picrossirius staining plus polarization microscopy, specific method of collagen detection in tissue section J Histochem 1979 11 447 455 10.1007/BF01002772 91593

[B27] Taboga SR Vidal BC Collagen fibers in human prostatic lesions: histochemistry and anisotropies J Submicrosc Cytol Pathol 2003 35 1 11 16 12762646

[B28] Fullmer HM Sheetz JH Narkates AJ Oxytalan connective tissue fibers: a review J Oral Pathol 1974 3 6 291 316 10.1111/j.1600-0714.1974.tb01724.x 4142890

[B29] Weibel ER Principles and Methods for the morphometric study of the lung and other organs Lab Invest 1978 12 131 155 13999512

[B30] Tsai M Shih LS Newlands GF Takeishi T Langley KE Zsebo KM Miller HR Geissler EN Galli SJ The rat c-kit ligand, stem cell factor, induces the development of connective tissue-type and mucosal mast cells in vivo. Analysis by anatomical distribution, histochemistry, and protease phenotype J Exp Med 1991 174 1 125 131 10.1084/jem.174.1.125 1711559 PMC2118877

[B31] Arizono N Kasugai T Yamada M Okada M Morimoto M Te H Newlands GF Miller HR Kitamura Y Infection of Nippostrongylus brasiliensis induces development of mucosal-type but not connective tissue-type mast cells in genetically mast cell-deficient Ws/Ws rats Blood 1993 81 10 2572 2578 7683922

[B32] Mecham RP Heuser JE Hay ED The elastic fiber Cell Biology of Extracellular Matrix 1991 2 New York: Plenum Press 79 106

[B33] Coplen DE Macarak EJ Howard PS Matrix synthesis by bladder smooth muscle cells is modulated by stretch frequency In Vitro Cell Dev Biol Anim 2003 39 3-4 157 162 10.1007/s11626-003-0010-3 14505431

[B34] Borges LF Caldini EG Battlhener CN Garcia SB Zucoloto S Montes GS Taboga SR Differential distribution of some extracellular matrix fibers in an experimentally denervated rat megaileum Micron 2008 39 4 397 404 10.1016/j.micron.2007.03.004 17433699

[B35] Hayward SW Rosen MA Cunha GR Stroma-epithelial interactions in the normal and neoplastic prostate Br J Urol 1997 79 18 26 9126066 10.1111/j.1464-410x.1997.tb16917.x

[B36] Ohno S Tachibana M Fujii T Ueda S Kubota H Nagasue N Role of stromal collagen in immunomodulation and prognosis of advanced gastric carcinoma Int J Cancer 2002 97 6 770 774 10.1002/ijc.10144 11857352

[B37] Vilamaior PSL Suzigan S Carvalho HF Taboga SR Structural characterization and distribution of elastic system fibers in the human prostate and some prostatic lesions Braz J Morphol Sci 2003 20 2 101 107

[B38] De Carvalho HF Vilamaior PS Taboga SR Elastic system of the rat ventral prostate and its modifications following orchiectomy Prostate 1997 32 1 27 34 10.1002/(SICI)1097-0045(19970615)32:1<27::AID-PROS4>3.0.CO;2-9 9207954

[B39] Vilamaior PS Felisbino SL Taboga SR Carvalho HF Collagen fiber reorganization in the rat ventral prostate following androgen deprivation: a possible role for smooth muscle cells Prostate 2000 45 3 253 258 10.1002/1097-0045(20001101)45:3<253::AID-PROS8>3.0.CO;2-P 11074528

[B40] Tracey KJ The inflammatory reflex Nature 2002 420 6917 853 859 10.1038/nature01321 12490958

[B41] Van Der Zanden EP Boeckxstaens GE De Jonge WJ The vagus nerve as a modulator of intestinal inflammation Neurogastroenterol Motil 2009 21 1 6 17 10.1111/j.1365-2982.2008.01252.x 19140954

[B42] Borovikova LV Ivanova S Zhang M Yang H Botchkina GI Watkins LR Wang H Abumrad N Eaton JW Tracey KJ Vagus nerve stimulation attenuates the systemic inflammatory response to endotoxin Nature 2000 405 6785 458 462 10.1038/35013070 10839541

[B43] Galvis G Lips KS Kummer W Expression of nicotinic acetylcholine receptors on murine alveolar macrophages J Mol Neurosci 2008 30 107 108 10.1385/JMN:30:1:107 17192650

[B44] Kawashima K Yoshikawa K Fujii YX Moriwaki Y Misawa H Expression and function of genes encoding cholinergic components in murine immune cells Life Sci 2007 80 24 25 10.1016/j.lfs.2007.02.036 17383684

[B45] Williams RM Berthoud HR Stead RH Vagal afferent nerve fibres contact mast cells in rat small intestinal mucosa Neuroimmunomodulation 1997 4 266 270 9650820 10.1159/000097346

[B46] Sudheer PS Hall JE Donev R Read G Rowbottom A Williams PE Nicotinic acetylcholine receptors on basophils and mast cells Anaesthesia 2006 61 1170 1174 10.1111/j.1365-2044.2006.04870.x 17090238

[B47] Tan TT Coussens LM Humoral immunity, inflammation and cancer Curr Opin Immunol 2007 19 209 216 10.1016/j.coi.2007.01.001 17276050

[B48] Mukherjee S Bandyopadhyay G Dutta C Bhattacharya A Karmakar R Baru IG Evaluation of endoscopic biopsy in gastric lesions with a special reference to the significance of mast cell density Indian J Pathol Microbiol 2009 52 1 20 24 10.4103/0377-4929.44956 19136773

[B49] Stead RH Colley EC Wang B Partosoedarso E Lin J Stanisz A Hillsley K Vagal influences over mast cells Auton Neurosci 2006 125 1-2 53 61 10.1016/j.autneu.2006.01.002 16500155

[B50] Heib V Becker M Taube C Stassen M Advances in the understanding of mast cell function Br J Haematol 2008 142 5 683 94 10.1111/j.1365-2141.2008.07244.x 18513284

[B51] Osinski MA Dahl JL Bass P Proliferation of mast cells in the smooth muscle of denervated rat jejunum J Auton Nerv Syst 1993 45 2 164 174 10.1016/0165-1838(93)90128-H 8282947

[B52] Samoszuk M Corwin MA Acceleration of tumor growth and peritumoral blood clotting by imatinib mesylate (Gleevec) Int J Cancer 2003 106 5 647 652 10.1002/ijc.11282 12866022

